# Neutron microtomography of voids in gold

**DOI:** 10.1016/j.mex.2017.11.009

**Published:** 2017-11-21

**Authors:** Pavel Trtik

**Affiliations:** Laboratory for Neutron Scattering and Imaging (LNS), Paul Scherrer Institut, 5232 Villigen PSI, Switzerland

**Keywords:** Neutron microtomography, Neutron imaging, Gold, Porosity

## Abstract

Pilot attempt of the neutron microtomography of voids in gold carried out using the Neutron Microscope instrument is presented in the paper. The paper demonstrates that neutron microtomography provides viable alternative to X-ray imaging for the assessment of porosity in high atomic number materials. The model sample based on gold with artificially induced void system reveals segmented porosity with 5.4 micrometres voxel size and the spatial resolution close to 10 micrometres.

## Highlights

• High-resolution microtomography of gold sample with artificially induced pore space.
• Comparison of the individual neutron radiograph with radiograph based on a common commercially-available tabletop X-ray source.
• Demonstration of potential for high resolution non-destructive quantification of porosity in other high atomic number materials (such as, precious metal alloys).

## Graphical abstract

**Figure:**
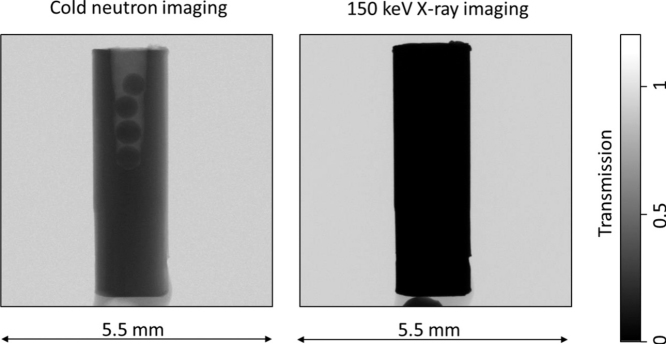

## Method details

Imaging with neutrons has been utilized for number of niche applications in which imaging with other types of radiation (such as X-rays) provides less convenient probe. One such example of niche application is imaging of microstructure of some very high atomic number materials [1], [2], [3], in which radiation in (hard) X-ray regime does not provide sufficient transmission signal to reveal the inner structure of such materials. The different contrast mechanisms of neutrons with matter (to that of X-rays) allow for investigation of materials based on high atomic number elements.

Objects made of high atomic number elements have been investigated using neutron imaging (i.e. tomography) already for some time [4]. It is, however, the recent progress in neutron imaging instrumentation (sub-10-μm domain) [5] that allows for imaging of representative volume elements of high atomic number materials with desired high spatial resolution.

In this paper, it is demonstrated that high-resolution neutron microtomography can readily reveal voids in high atomic number materials with high spatial resolution in (for neutron imaging) affordable acquisition time. For the purpose of this demonstration, a sample of gold with artificially induced porosity has been prepared and investigated at the Paul Scherrer Institut, at ICON [6] and BOA beamlines [7] using the Neutron Microscope instrument [5], [8].

## Sample preparation and test arrangement

In order to demonstrate the feasibility of high-resolution neutron microtomography of porosity in high atomic number materials, a sample of gold with artificial void system has been produced in the following manner. First, a small gold rod of 1.6 mm in diameter and of approximately 5 mm in length was cut from a longer piece of commercially available high-purity gold rod. A small hole – of about 2.5 mm in depth– was drilled into one side of the rod using a 0.5 mm drill. The resulting hole was filled with four commercially available gold spheres of 0.5 mm in diameter. The sample has been then imaged at the ICON beamline [6] using first high-energy X-rays and later neutrons. Both X-rays and neutron radiation has been detected using approximately 3.5 μm thick isotopically-enriched 157-gadolinium oxysulfide scintillator screen [9]. The resulting visible light was magnified by high-numerical aperture optics and collected using a high-performance CCD detector. The nominal pixel size of the radiographic images taken at ICON beamline was equal to 2.7 μm and the full field of view of 2048 × 2048 pixels was thus equal to approximately 5.5 × 5.5 mm × mm. A commercially-available table-top X-ray source provided high-energy X-rays and was set to 150 keV accelerating voltage and 500 mA beam current. The distance between the X-ray source and the scintillator screen was approximately 400 mm. The neutron radiographs were taken using the full white beam available at the ICON beamline. The size of the beam defining aperture was equal to 20 mm in diameter and the distance between the detector and the aperture was about 8.7 m, leading to L/D ratio of about 435. Stacks of individual projections of 60 s acquisition time were taken both for X-ray and neutrons.

After that the Neutron Microscope instrument and the small gold sample was transferred to BOA beamline [7], where beamtime availability allowed for a microtomography experiment to be performed. During the transfer, the top most gold sphere fell out of the sample and therefore the sample with three gold spheres only was tomographed using the following test arrangement. The size of the beam defining aperture was equal to 40 × 40 mm × mm and the distance between the detector and the aperture was about 5.5 m, leading to L/D ratio of about 138. The mean distance of the sample and the scintillators screen was equal to about 1 mm.

Projections images were acquired in 225 angular positions spread uniformly over 360 ° travel range (step size 1.6°). In each angular position 11 individual radiographs of 60 s exposure time were taken. For the purpose of affordable acquisition time, 2 × 2 pixel binning was used directly at the detector, leading to a pixel size of 5.4 μm and the full field of view of 1024 × 1024 pixels.

In order to suppress the white spots in the images, the resulting radiograph in each angular position was obtained as a median filtered image of all the 11 projections. Thus, 2486 individual projections were taken over the course of about 5 days of experimental time. However, it needs to be highlighted that the experiment has been halted in the process of the acquisition by unavailability of the neutrons during a single beam-down lasting approximately 3 days. The open beam image was based on 200 individual images of 60 s exposure time. In total, the necessary exposure time for the experiment has been approximately 2 days.

Afterwards, the standard filtered backprojection algorithm (Parzen convolution filter, cut-off frequency = 1.0) was used for the reconstruction of the microtomgoraphic dataset. Combined wavelet-Fourier stripe filtering algorithm [10] was used for suppression of the ring artifacts in the sinogram regime. In the last step, 3D anisotropic diffusion filter developed by Münch [11] [12], was used as the edge preserving filter for the dataset.

## Results and discussion

**Fig. 1:**
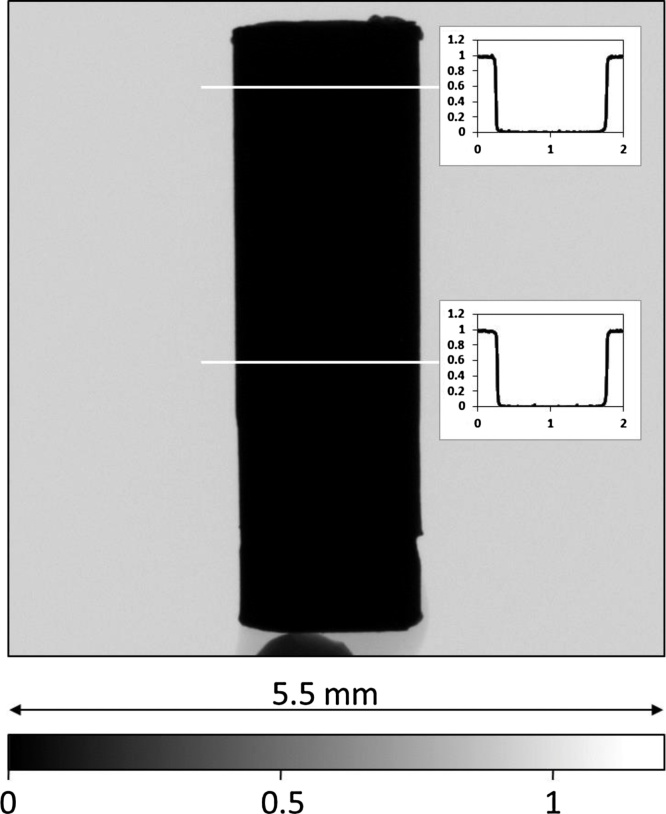
X-ray radiograph of the gold sample with artificial pore space. The inserted transmission profiles confirm that hardly any X-rays are transmitted through the sample.

Fig. 1 presents the X-ray radiograph of the sample that is based on median filter of three sample projections and three open beam images. After the correction for background, it is apparent that the sample of gold is nearly fully opaque for up to 150 keV X-rays. The theoretically calculated transmission for the given samples thickness for 150 keV X-rays is in the order of 10^−7^, therefore the transmission values close to zero were acquired even for the very thin parts of the sample). The image clearly demonstrates that much higher energies of X-rays than those available by commercially available table-top X-ray sources would be required for successful X-ray tomography of such samples.

**Fig. 2:**
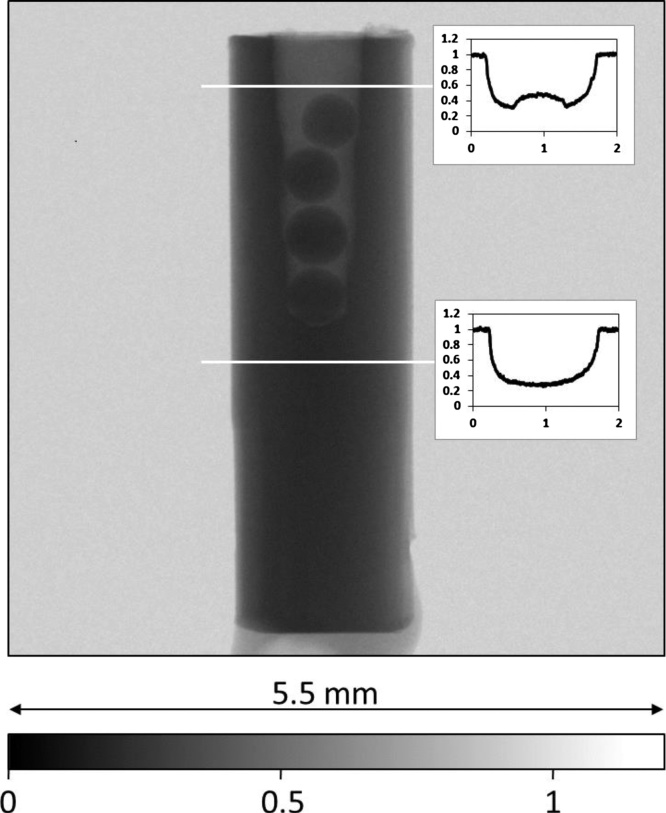
Corresponding neutron radiograph of the gold sample with artificial voids. The artificial pore space formed by 0.5 mm gold spheres placed in the drilled hole. The inserted transmission profiles clearly show clearly that inner pore space of the gold sample can be clearly revealed using neutrons.

Fig. 2 shows the corresponding radiograph based on cold neutrons. The resulting image is based on 48 individual projections of the sample and 48 open beam projections of 60 s exposure time. Even in 2D, the radiograph clearly reveals the tapering shape of the drilled axial hole, as well as the position of the gold spheres and the artificial voids formed by the walls of the drilled hole and the spheres.

**Fig. 3:**
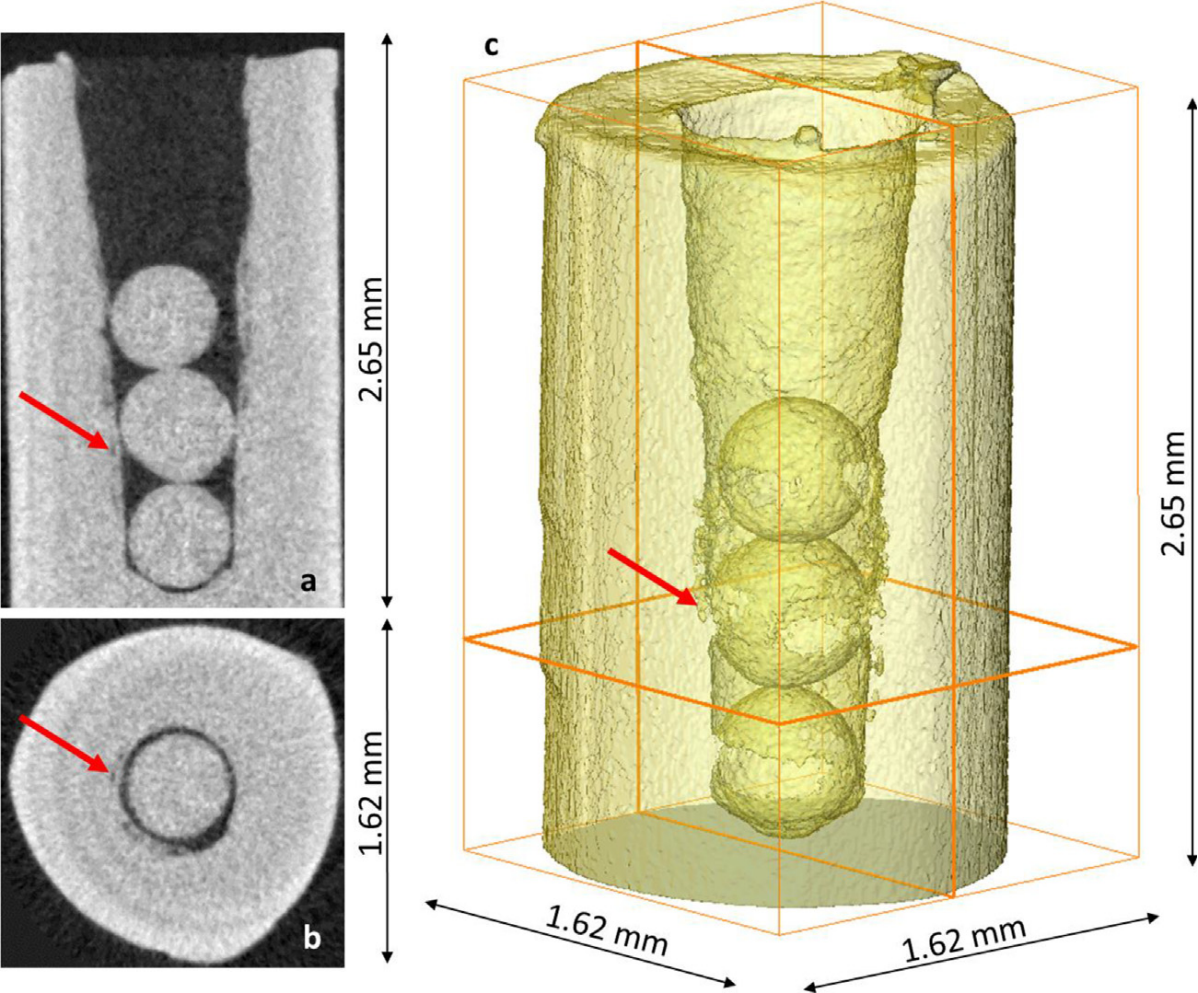
(a) vertical and (b) horizontal slice of the reconstructed neutron microtomographic dataset revealing clearly the porosity in the gold sample with high resolution. Voxel size of the dataset equals 5.4 μm. (c) 3D partially transparent rendering of the sample clearly revealing the artificial pore space of the sample. Positions of the horizontal and vertical slices shown in Figs. 3a and ab are clearly indicated. Examples of the revealed porosity occurring in the close vicinity of the drilled hole is indicated by the red arrows in all three images. (For interpretation of the references to colour in this figure legend, the reader is referred to the web version of this article.)

Fig. 3a and 3b show a vertical and a horizontal slices of the reconstructed microtomographic dataset. There are several additional features that can be clearly observed from the dataset.

First, it is clearly shown that the originally cylindrical shape of the sample has been deformed during the sample preparation by fixing the sample for drilling in a collet (see the outer sample shape in Fig. 3b). Second and more important, additional porosity in the close vicinity (of several tens of micrometres) from the drilled hole has been revealed (see Fig. 3a and 3b) and could be even segmented in 3D (see Fig. 3c). The size of those artificially induced voids is in the order of ∼10 μm. Likewise, the size of the voids that can be trustworthily segmented in the regions of the contacts of the individual spheres of the diameter of 0.5 mm is about 10 μm.

The resolution can be surely further improved to the limit of the Neutron Microscope instrument by utilizing the imaging without image binning. However, such microtomography experiment would – for this test arrangement at the BOA beamline- need to last for unreasonable time (few weeks). At the same time, it has to be highlighted that BOA beamline – while providing good contrast due to the available cold spectrum– does not offer the highest available neutron flux [7]. Therefore, even higher spatial resolution similar experiments are foreseen to be performed at PSI at other beamlines (e.g. POLDI), or at other high-flux neutron sources (such as ESS, ILL, MLZ, etc.). From the point of view of data reconstructions, it is foreseen that the superior temporal resolution of microtomography can be achieved by sinogram inpainting [13] and even higher spatial resolution can be possibly attained by subvoxel precision registration of the projection set [14].

Regarding the future scientific/industrial applications for the Neutron Microscope instrument, the author trusts that neutron microtomography might be of interest to the material science of precious metal alloys (e.g. [15], [16], etc.).

## Conclusion

The paper demonstrates that neutron microtomography provides viable alternative to X-ray imaging for the assessment of porosity in high atomic number materials (such as gold). The model sample based on gold with artificially induced void system reveals segmented porosity with 5.4 micrometres voxel size and the spatial resolution is assessed to be close to 10 micrometres.
